# The polyene antifungal candicidin is selectively packaged into membrane vesicles in *Streptomyces* S4

**DOI:** 10.1007/s00203-022-02906-w

**Published:** 2022-04-30

**Authors:** Sarah A. Blackburn, Mark Shepherd, Gary K. Robinson

**Affiliations:** School of Biosciences, Division of Natural Sciences, University of Kent, Canterbury, CT2 7NJ UK

**Keywords:** Streptomyces, Gram-positive, Membrane vesicles, Antifungal, Antimycin, Candicidin

## Abstract

In recent years, much attention has been focused on the biogenesis, engineering and utilisation of outer membrane vesicles (OMVs) in Gram-negative bacteria in a range of environments and niches. While the precise mechanism of biogenesis is unknown, it is focused on the modification of the Gram-negative cell wall to facilitate blebbing at sites of weakness in and around the characteristically thin peptidoglycan layer within the periplasm. Here, we investigate the biogenesis of membrane vesicles (MVs) in the Gram-positive organism *Streptomyces albus* S4 (Seipke et al. J Bacteriol 193:4270–4271, 2011 and Fazal et al. Antonie Van Leeuwenhoek 113:511–520, 2020). The *S. albus* S4 strain is an antifungal (candicidin and antimycin) producing organism that was isolated from attine ants (Barke et al. BMC Biol 8:109, 2010). The biogenesis and characterisation of *S. albus* S4 MVs is demonstrated using the wild-type (WT) and mutant strains *ΔantC* (no antimycin production) *ΔfscC* (no candicidin production) and *ΔantC* Δ*fscC* (produces neither antimycin nor candicidin). Here, we have shown that the *S. albus* S4 strain produces MVs and that these are comprised of both specific protein profiles and secondary metabolites, with a clear demonstration of the ability to selectively package one antifungal (candicidin) but not the other (antimycin).

**Supplementary Information:**

The online version contains supplementary material available at 10.1007/s00203-022-02906-w.

## Introduction

Outer membrane vesicles (OMVs) are nano-sized, spherical vesicles, which are formed from the outer membrane of Gram-negative bacteria. OMVs have been the focus of much research since their original discovery by electron microscopy in the 1960 s (Chatterjee and Das 1966; Work et al. 1966). They are approximately 20–250 nm in diameter (Schwechheimer and Kuehn 2015) and can contain a wide range of cargo, which travel within the OMVs to reach their target in a concentrated and protected form. OMV cargo often includes virulence factors such as toxins or proteases, which are beneficial to the bacteria that secrete them. One of the main components of bacterial cell walls is the peptidoglycan layer, which preserves the integrity of the cell and helps to prevent cell lysis (Silhavy et al. 2010). Evidence suggests that OMVs are formed at sites where the lipoprotein (Lpp) links between the peptidoglycan layer and OM are missing or broken, although, the exact mechanism is not fully understood (Avila-Calderón et al. 2021).

One of the main differences between Gram-positive and Gram-negative cell envelopes is the thickness of the peptidoglycan layer and the possession of an oxidising and defined periplasmic compartment in the latter. Gram-positive bacteria have a relatively thick peptidoglycan layer of 20–80 nm in diameter, compared to < 10 nm in Gram-negative bacteria (Mai-Prochnow et al. 2016). More recent evidence in the literature suggests that Gram-positive bacteria also produce membrane vesicles (MVs), despite the presence of a thick peptidoglycan layer (summarised in Liu et al. 2018 and Nagakubo et al. 2020). These are different to OMVs in cargo and composition due to differences in the membrane structure (Brown et al. 2015; Bitto et al. 2017). OMVs derived from Gram-negative bacteria are composed of the outer membrane and contain cargo from the periplasm. Gram-positive MVs, however, are composed of the cytoplasmic membrane only and cargo from the cytosol (Joffe et al. 2016).

The exact mechanism of how MV biogenesis occurs in Gram-positive bacteria is still unknown; however, there are a range of non-mutually exclusive hypotheses. One hypothesis is that the turgor pressure produced after MVs are released from the cytoplasmic membrane is enough to force the MVs through the peptidoglycan cell wall. It is possible that there are pore sizes within the cell wall that are large enough to allow the MVs to pass through (Brown et al. 2015). It has also been proposed that peptidoglycan-degrading enzymes are released to degrade areas of the peptidoglycan layer to allow MV formation and secretion. Examples of these enzymes include endolysins in *Bacillus subtilis* (Toyofuku et al. 2017) and autolysins in *Staphylococcus aureus* (Wang et al. 2018). One final hypothesis is that MVs are transported through the peptidoglycan layer using protein channels. This would allow the passage of MVs through the peptidoglycan layer without disrupting the cell wall (Brown et al. 2015). The current hypotheses for Gram-positive MV biogenesis have been discussed and summarised in Brown et al., (2015), Toyofuku et al. (2017), Wang et al. (2018) and Nagakubo et al. (2020).

In 2009, a study reported that membrane vesicles had been visualised on the surface of *Staphylococcus aureus* cells using TEM (Lee et al. 2009). The MVs were very similar in appearance to archetypal OMVs as they were nano-sized, spherical membranous structures of 20–100 nm in diameter. The purified MVs were visualised by TEM and the size of the MVs were determined using dynamic light scattering and characterised using proteomics (including SDS-PAGE, western blotting and Nano-LC-ESI-MS/MS0). MV proteomes have been found to contain cytoplasmic proteins, metabolic enzymes, DNA polymerases, ribosomal proteins and virulence factors (Joffe et al. 2016.) Gram-positive cells also appear to have a specific sorting mechanism for packaging cargo into the secreted MVs. For example, in 2010, MVs from *Bacillus anthracis* were found to contain a relatively high concentration of anthrax toxin components (i.e., edema factor, lethal factor and protective antigen), which were not present freely in the supernatant (Rivera et al. 2010).

Of particular relevance to the present work has been the demonstration of MV production in specific Actinobacteria, notably *Streptomyces coelicolor* (Schrempf et al. 2011 and Faddetta et al. 2021), *Streptomyces lividans* (Schrempf and Merling 2015) and *Streptomyces* Sp. Mg1 (Hoefler et al., 2017). *Streptomyces* are filamentous soil bacteria that produce the majority of antibiotics used for medicine, agriculture and veterinary practice (Chater, 2016). In the previous publications, it was shown that the *Streptomyces* spp. produced visible exudates that were enriched in MVs and contained a plethora of proteins, lipids and bioactive cargoes such as actinorhodin (a benzoisochromanequinone dimer polyketide antibiotic produced by *S. coelicolor*) and undecylprodigiosin (an alkaloid produced by *S. coelicolor* and *S. lividans*). The comprehensive audit of *S. coelicolor* MVs by Faddetta et al. (2021) showed both an extensive array of 166 proteins (65 shown to be luminal) and a diverse small molecule metabolome containing bioactives such as actinorhodin and undecylprodigiosin. Aside from characterising the yield and content of *Streptomyces*’ MVs, the study by Hoefler et al. (2017) was key to our studies having shown that a specific polyene antibiotic (linearmycin) was packaged in MVs and that disruption of its synthesis (Δ*lnyI*) significantly depleted the yield of MVs.

In the present study, MVs were isolated and characterised from *Streptomyces albus* S4 (details in Barke et al. 2010, Seipke et al. 2011 and Fazal et al. 2020), which have a symbiotic relationship with the leaf-cutting ants *Acromyrmex octospinosus*. These ants have evolved to cultivate fungi in specialised chambers in their nests to use as a food source (Haeder et al. 2009). The antibiotics that Actinobacteria (such as *Streptomyces*) produce are thought to help keep these fungal gardens free of other unwanted microbes, protecting their food source. The *Streptomyces* S4 genome contains biosynthetic gene clusters which lead to the synthesis of the antifungal compounds candicidin (Barke et al. 2010) and antimycin (Seipke et al. 2011). Candicidin is a polyene antifungal that binds to ergosterol, affecting membrane permeability and integrity*.* This leads to a rapid efflux of K^+^ ions and ultimately cell death. The antimycins are a group of compounds that inhibit cytochrome c reductase, an essential enzyme in the mitochondrial electron transport chain. Inhibition of this enzyme disrupts the entire electron transport chain and inhibits cellular respiration which leads to cell death. Both antifungals are known to be toxic towards a range of pathogenic fungi including *Candida albicans* (Barke et al. 2010) and are, therefore, of medical relevance. Although previous evidence has found that *S. albus* S4 produces both candicidin and antimycin antifungals, it is currently not known how they are secreted extracellularly. This was investigated in this study using the *S. albus* S4 wild-type (WT) strain and mutant strains *ΔantC* (no antimycin production) *ΔfscC* (no candicidin production) and *ΔantC* Δ*fscC* (produces neither antimycin nor candicidin). Further details can be found in Seipke et al. (2011) and Supplementary Information 1.

AmBisome^®^ is a drug delivery system used to treat infections from a wide range of fungal pathogens (Stone et al. 2016). AmBisome^®^ uses a liposome (an artificially made spherical vesicle formed of a phospholipid bilayer) to target a fungal pathogen (such as *C. albicans*) and deliver the antifungal compound amphotericin B across the cell wall. The liposomes preferentially adhere to the fungal cell wall and then release the active amphotericin B molecule which binds to ergosterol in the fungal cell membrane. These pores alter cell permeability and lead to the leakage of monovalent ions (Cl^−^, H^+^, K^+^ and Na^+^) and cell death (Walker et al. 2018). Due to the known success of the delivery of amphotericin B within a liposome to fungal cells, we reasoned that *S. albus* S4 may naturally produce ‘AmBisome^®^-like’ membrane vesicles to allow delivery of active antifungal molecules (candicidin and/or antimycin) in a targeted and efficient way. The present study provides strong evidence that *S. albus* S4 secretes membrane vesicles that specifically contain the polyene antifungal candicidin but not antimycin, which are concentrated for release into the environment.

## Results

- *Streptomyces albus* S4 WT bacteria secrete membrane vesicles that can be visualised using TEM and confocal microscopy

**Fig. 1:**
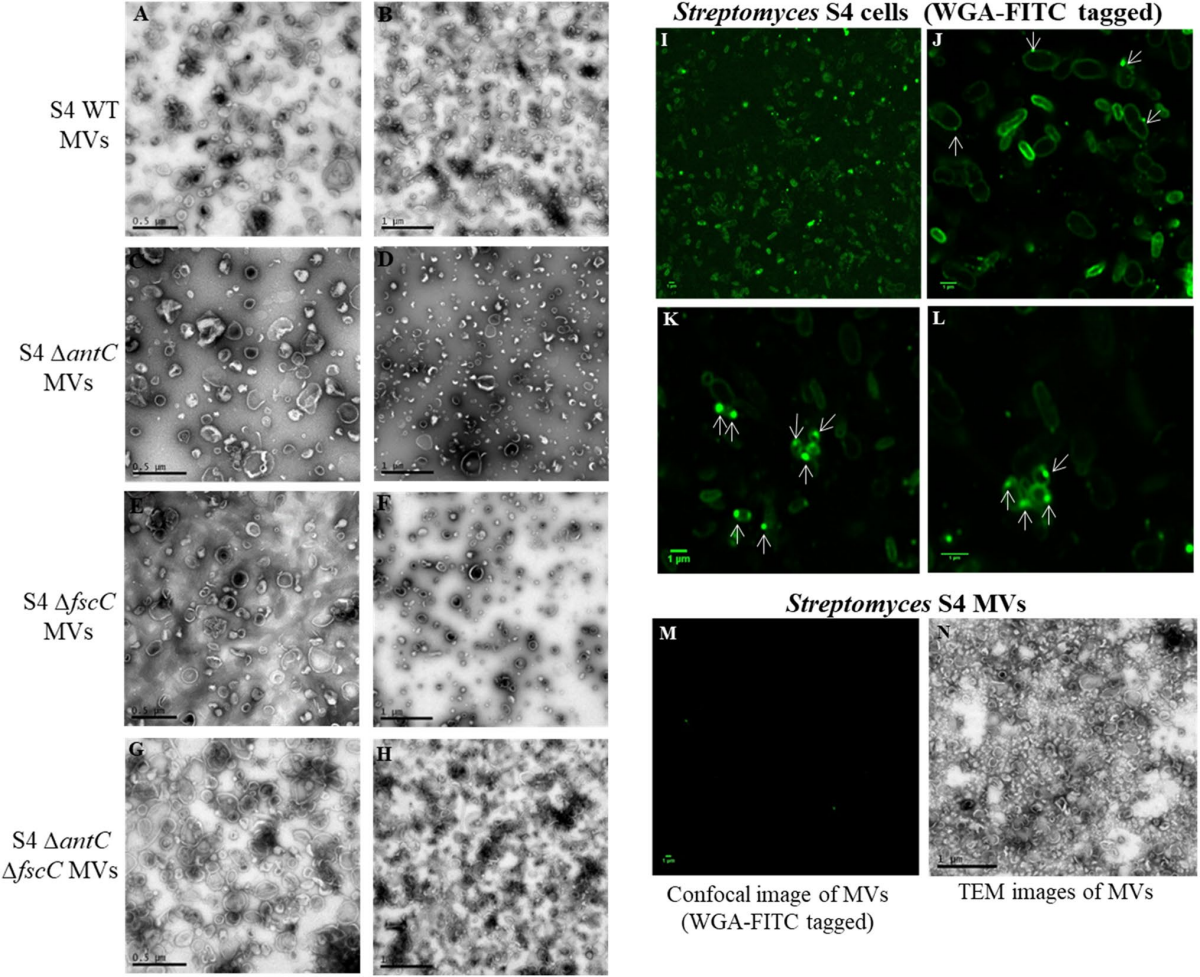
Visualisation of membrane vesicles from *Streptomyces albus* S4 strains Purified MVs were visualised using TEM from the following *S. albus* S4 strains: wild type (WT) (**A**, **B**), *ΔantC* (**C**, **D**)*, ΔfscC* (E–F) and *ΔantC ΔfscC* (**G**, **H**)*. S. albus* S4 WT cells were fixed in 2% (w/v) formaldehyde then incubated in 100 µg/mL WGA-FITC. Samples were visualised the next day by confocal microscopy (**I**, **L**). Images **I**, **L** show increasing magnifications of the same sample and the white arrows are used to indicate potential MVs budding from the *Streptomyces* cells (**J**, **L**)*.* All images (**I**, **L**) show a 1 µm scale bar. Lastly, purified MVs from *S. albus* S4 WT strain were fixed in 2% (w/v) formaldehyde and incubated in 100 µg/mL WGA-FITC. (M) As a comparison, the same sample of *S. albus* S4 WT MVs were fixed and photos were taken using TEM for a direct comparison (N)

An OMV purification protocol used previously with *E. coli* (Blackburn et al. 2021), was applied to *Streptomyces* S4 WT and the three mutant strains to see if any MVs were produced and if they could be isolated for characterisation. The resulting samples were concentrated (50x) then visualised using TEM and photos were taken at various magnifications (Fig. 1A–H). The TEM images demonstrated that MVs had successfully been purified from all four *S. albus* S4 strains and that the *ΔantC* and *ΔfscC* mutations did not appear to adversely impact upon the MV yield or size distribution. Additionally, the purified MVs were very similar in appearance to OMVs previously purified from *E. coli* and *P. aeruginosa* (Supplementary Information 2) and also MVs that have previously been isolated from *Streptomyces coelicolor* (Schrempf et al. 2011), *Streptomyces lividans* (Schrempf et al*.* 2015) and *Streptomyces venezuelae* (Fröjd et al*.* 2019).

The Gram-positive cell wall is enriched in peptidoglycan and the proposed mechanisms for the biogenesis of MVs state that the cytoplasmic membrane blebs at sites of peptidoglycan weakness (Brown et al. 2015). We reasoned that peptidoglycan from the cell wall should be present on *Streptomyces* whole cells but may not be present on the surface of the secreted MVs. We sought to probe this using wheat germ agglutinin (WGA), a lectin from *Triticum vulgaris* (wheat) that binds to the N-acetyl glucosamine present in peptidoglycan. The WGA is conjugated to the fluorescent conjugate fluorescein isothiocyanate (FITC) to allow visualisation by confocal microscopy. *S. albus* S4 cells and MVs were visualised using WGA-FITC (Fig. 1I–M) based on a protocol found in the literature (Celler et al. 2016).

*S. albus* S4 cells were harvested from cells growing in stationary phase and were treated with WGA-FITC as outlined in Materials and methods. As expected, WGA-FITC successfully bound to the peptidoglycan layer in the whole cells (Fig. 1I–L). Intriguingly, some areas of intense fluorescence were also found on the membranes of the cells, which may indicate the sites of vesiculation (white arrows on Fig. 1J–L). Purified MVs from each *Streptomyces* strain (WT, Δ*antC*, Δ*fscC* and Δ*antC* Δ*fscC*) were concentrated (50x) and incubated with WGA-FITC using the same method previously used for the whole cells (Fig. 1M and Supplementary Information 3). An example of the staining is shown alongside a TEM image of the same WT MV sample (Fig. 1M, N). The TEM image showed that the MV sample was very concentrated and dense with purified MVs (Fig. 1N). However, any areas of fluorescence were rare and very dispersed when present (Fig. 1M), suggesting that the secreted MVs are composed of the cytoplasmic membrane only and do not contain peptidoglycan (or glycoproteins containing β (1 → 4)-N-acetyl-D-glucosamine) on their surface.

b) Analysis of the MV-associated proteins purified from *Streptomyces albus* S4

**Fig. 2:**
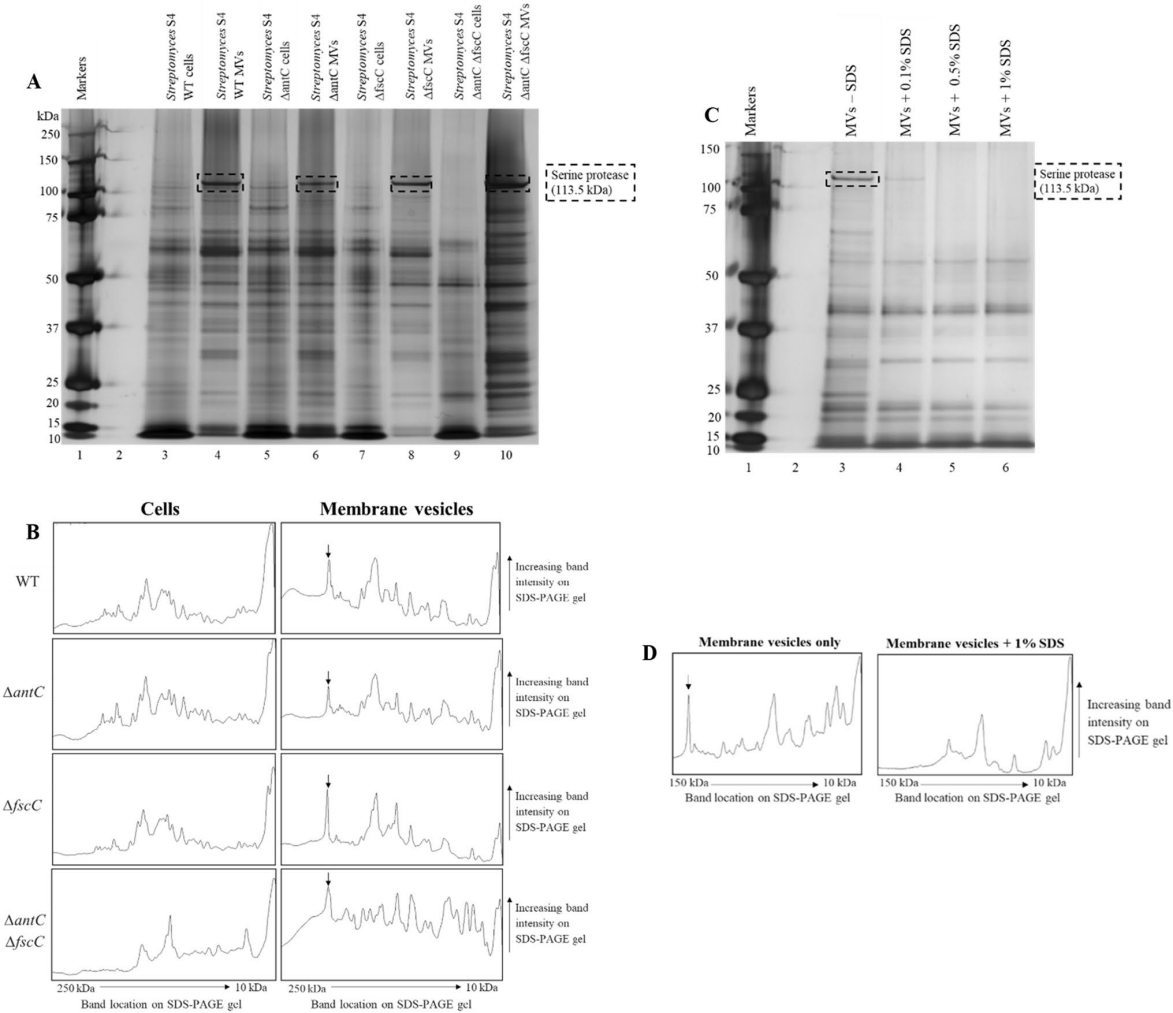
Comparison of the MV protein profile from *Streptomyces albus* S4 and the 3 mutant strains *(∆antC, ∆fscC* and *∆antC ∆fscC*) MVs were purified from four different *S. albus* S4 strains: WT, ∆*antC,* ∆*fscC* and ∆*antC* ∆*fscC* and the protein profiles were compared to that of the whole cell samples. All samples were standardised to be the same protein concentration and concentrated using TCA precipitation. An SDS-PAGE gel was run then silver stained to visualise the cell and MV protein profile (**A**). *S. albus* S4 WT MVs were incubated with various concentrations of SDS (0.1–1%) at 37 °C for 60 min. The samples were run on an SDS-PAGE gel then silver stained to visualise the MV protein profile (**C**). Protein densitometry plots were generated from the SDS-PAGE gel photos using Fiji (Image J). The plots generated relate to the density/intensity of the bands in each lane and the arrows indicate the serine protease at 113.5 kDa (**B**, **D**)

We have shown previously that the OMVs of Gram-negative bacteria have an enrichment of specific proteins within the vesicles when compared with whole cells and subcellular fractions (Blackburn et al. 2021). We wanted to investigate whether this was mirrored in a Gram-positive organism, accepting that the specific biogenesis of the MV was likely to differ markedly. The protein profile of both the whole cells and MVs purified from the four *S. albus* S4 strains was compared and this is shown in (Fig. 2A, B). On initial inspection, it can be concluded that the proteins present within the MVs differed markedly from their originator cells (e.g. Lane 3 vs Lane 4 for WT sample). It was also concluded that, while the proteins present in the MVs derived from *S. albus* S4 WT (Lane 4), ∆*antC* (Lane 6) and ∆*fscC* (Lane 8) appeared the same, the protein profile of ∆*antC* ∆*fscC* appears to contain extra proteins (Lane 10). Perhaps, the regulation of which proteins enter the MVs was disrupted when the cell no longer produces either antimycin or candicidin.

From Fig. 2A, B, it is clear that there are MV specific proteins, i.e. proteins that appear to be absent or at low concentrations in the whole cell preparations but appear to be dominant in the MV protein profile. This was best exemplified by the band at approximately 110–120 kDa, which was identified as a serine protease (113.4 kDa) (see Supplementary Information 4). In previous OMV characterisation work, *E. coli* OMVs were incubated with the detergent SDS, which appeared to cause disruption to the membrane of the OMVs and release of active proteases from within (data not shown). These proteases degraded some of the OMV-associated proteins (and flagella) and changed the OMV protein profile. This method was applied to purified *S. albus* S4 WT MVs to see if they contain active proteases (Fig. 2C, D). *S. albus* S4 WT MVs were purified and incubated with increasing concentrations of SDS and the MV protein profile was visualised using SDS-PAGE and silver staining of the gel. It was found that many of the bands disappeared when various concentrations of SDS were added (Lanes 4–6 compared to Lane 3). This indicates that proteases were present and were released when the membrane is disrupted by SDS. One of these proteases may be the serine protease detected by mass spectrometry.

c) Investigating the non-protein cargo of MVs derived from *Streptomyces* S4

*S. albus* S4 and the relevant mutant strains (∆*antC,* ∆*fscC* and ∆*antC,* ∆*fscC*) were chosen to investigate whether the antifungals produced by the bacterial cells were packaged into the secreted MVs. This hypothesis was initially formulated based on AmBisome®, the synthetic liposomal amphotericin B formulation that has been shown to cross the viscoelastic cell wall in both *Candidia albicans* and *Cryptococcus neoformans* (Walker et al. 2018). Candicidin is a non-water-soluble polyene antifungal antibiotic, that was already known to be produced by *S. albus* S4 and gave a large zone of inhibition when plated on a lawn of *C. albicans* (Barke et al. 2010). We reasoned that dissemination of candicidin did not rely on simple diffusion for packaging and secretion into the environment by the *S. albus* S4 cells. To determine if antifungal compounds (such as candicidin) were packaged into *S. albus* S4 MVs, a bioassay method to observe the effect on the growth of *C. albicans* was developed. This was initially trialled with various concentrations of authentic candicidin and antimycin ranging from 1 to 200 µg/mL (see Materials & Methods for more details). The results are shown in Supplementary Information 5 where it was clearly shown that the candicidin was inhibitory at > 10 µg/ml and antimycin at > 5 µg/ml i.e., antimycin had double the potency of candicidin in this assay.

**Fig. 3:**
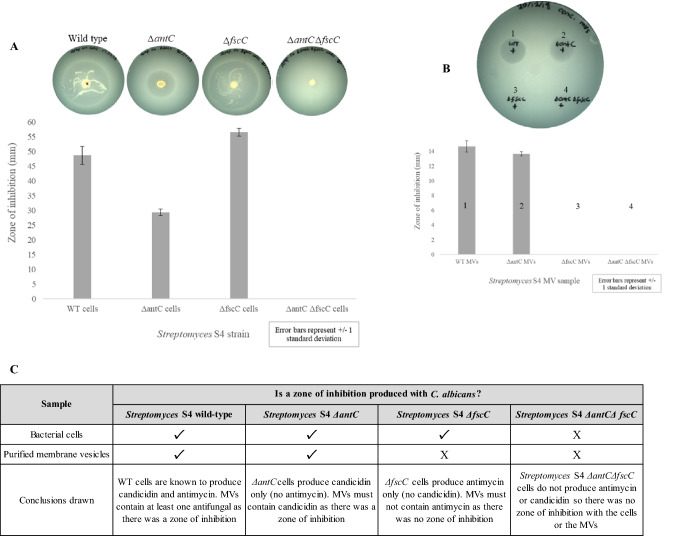
The effect of *Streptomyces albus* S4 cells and purified MVs on *Candida albicans* growth *S. albus* S4 colonies (grown on MS plates) were inoculated into TSB/YEME media and incubated for 72 h at 30 °C. 5 μL of this culture was spotted at the centre of a LB agar plate and left to soak/dry for 2 h. The plates were incubated at 30 °C for 72 h to allow the growth of *Streptomyces*. A liquid culture of *C. albicans* (grown in YPD at 37 °C overnight) was mixed with LB 0.5% (w/v) agar then overlayed on to the plates and left to set. Plates were incubated at 37 °C for 18 h and the diameter of the zone of inhibition was measured (**A**). 5 μL 25 × concentrated MVs were added to the LB plates and left to soak/dry for 2 h at room temperature. *C. albicans* mixed with LB 0.5% (w/v) agar was overlayed on to the plates and left to set. Plates were incubated at 37 °C for 18 h and the diameter of the zone of inhibition was measured (**B**). All plates were prepared in triplicate and the average zone of inhibition was calculated and presented as a graph. These findings are summarised as a table (**C**)

Having established the bioassay with candicidin and antimycin standards, we then tested the strains (WT, Δ*antC*, Δ*fscC* and Δ*antC* Δ*fscC*) and purified MVs to observe the zones of inhibition produced when plated on a lawn of *C. albicans*. As expected, zones of inhibition were produced from *S. albus* S4 WT, *∆antC* and *∆fscC* whole cells but not from the *∆antC ∆fscC* strain (Fig. 3A). MVs purified from the four strains were concentrated (25 × by TCA precipitation) and subject to the same bioassay. The results of (Fig. 3B, C) indicate that *S. albus* S4 MVs contained the antifungal compound candicidin but not antimycin, which is a novel finding. Interestingly, when the individual mutants are compared, it was shown that *ΔantC* (candicidin producer) was less inhibitory than Δ*fscC* (antimycin producer) when whole cells were tested (Fig. 3A) but this effect was not retained when the MVs were tested (Fig. 3B). This strongly suggested that candicidin is actively packaged into the MVs but antimycin is excluded (summarised in Fig. 3C).

A further experiment was conducted to see whether concentrating or washing the MVs with HEPES buffer modulated their antifungal activity (Supplementary Information 6). While the overall findings were the same as shown previously, it was additionally demonstrated that washing the MV pellets had a negligible effect. Moreover, concentration of the MVs (25x) did not linearly amplify potency as there was only a 50% increase in the zone of inhibition. This suggested that there are other physicochemical factors that influence the diffusion and antifungal potency of candicidin from the vesicles. Lastly, to find further evidence that candicidin is MV-associated, *S. albus* S4 MVs were washed 1 × , 2 × and 3 × with HEPES buffer. This was to remove any extracellular material from around the MVs so that any antifungal activity observed was from MVs only (Supplementary Information 7). This provided further evidence that the zones of inhibition found for the MVs after two or three washes in HEPES buffer is MV-associated as the antifungal activity remained.

d) Confirmation that candicidin is membrane vesicle (MV) associated in *Streptomyces albus* S4

**Fig. 4:**
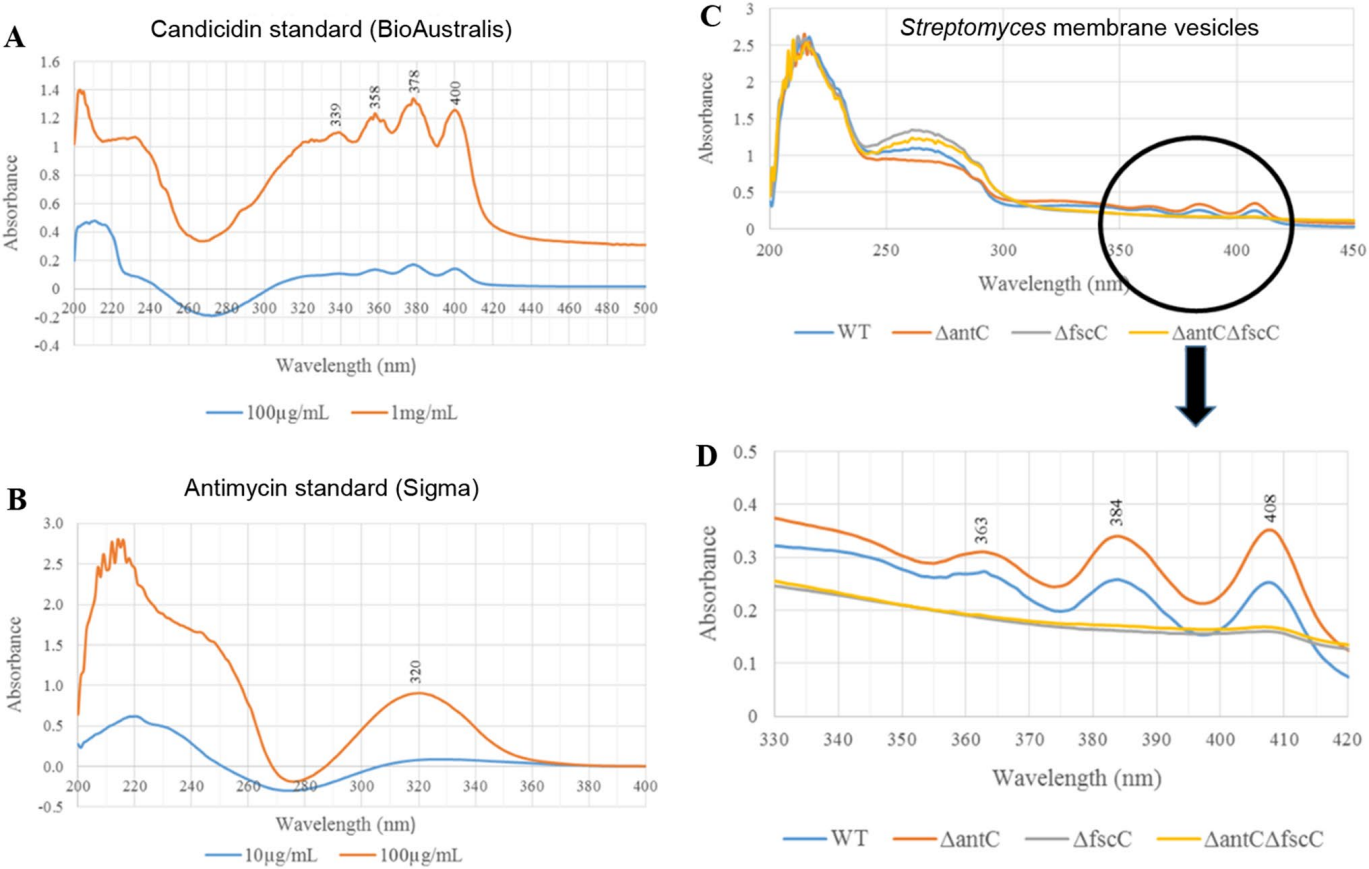
UV–Vis spectra of candicidin detected in *Streptomyces albus* S4 MVs Authentic candicidin and antimycin were dissolved at 1 mg/mL and 100 µg/mL in absolute ethanol and UV–Vis spectra were obtained using Agilent Technologies Cary 60 UV–Vis spectrophotometer. The peaks that are ‘characteristic’ of each antifungal compound are labelled in (**A**, **B)**. *S. albus* S4 MVs were purified using the standard protocol but the MV pellets were resuspended directly into butanol. MVs (extracted in butanol) were run on the spectrophotometer (as above) to obtain the UV–Vis spectra (**C**) and the area of interest was enlarged to see the characteristic candicidin peaks (**D**)

**Fig. 5:**
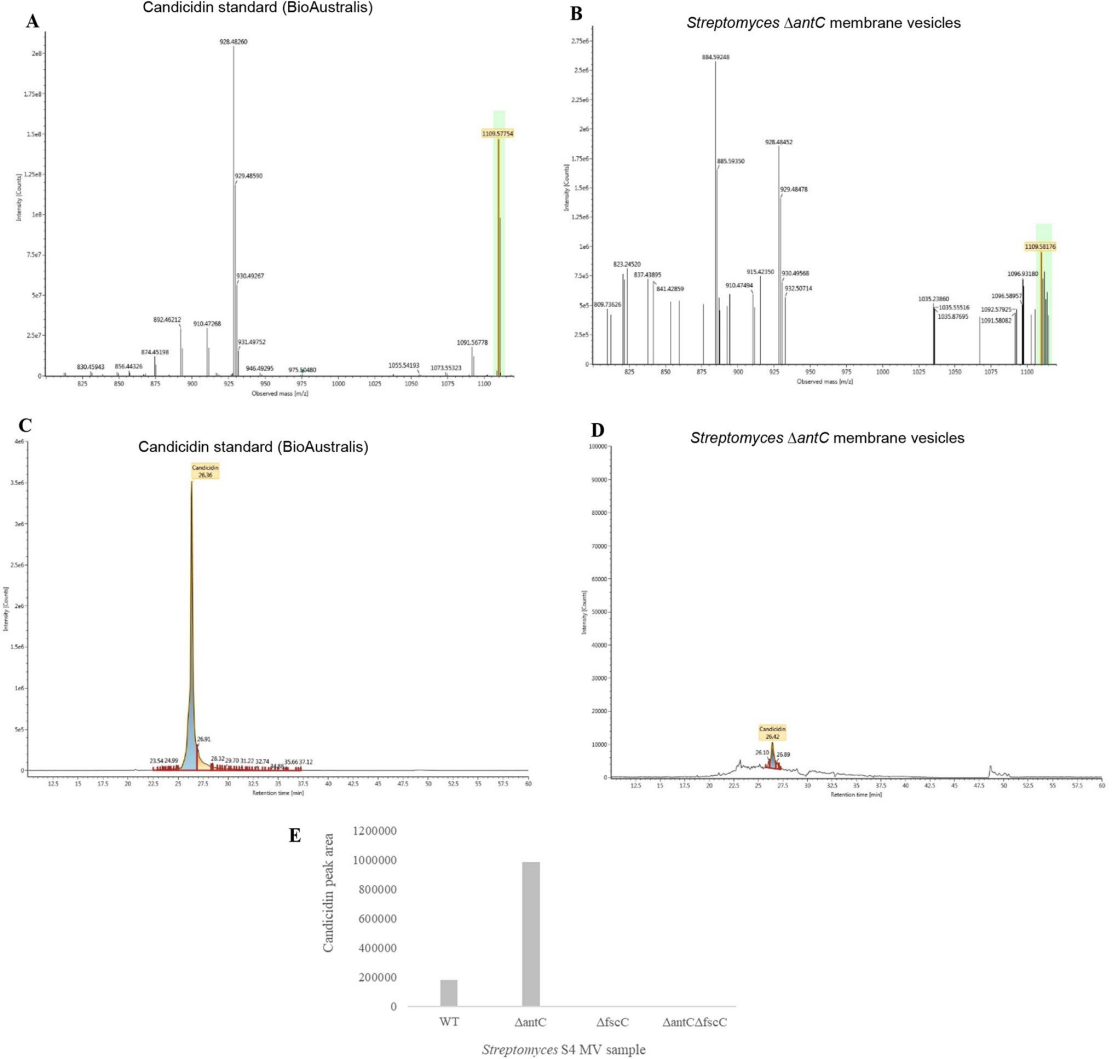
Confirmation of candicidin present in *Streptomyces albus* S4 membrane vesicles using mass spectrometry Purified vesicles were analysed by ultra-performance nanoLC (ACQUITY M Class) coupled to an IMS mass spectrometer (SYNAPT G2-Si, Waters) fitted with a NanoLockSpray source. The data was processed using the UNIFI software. Extracted ion chromatograms were produced to select for the detection of candicidin (EIC [M + H]^+^, 1109.5720 m/z) and antimycin (EIC [M + H]^+^, 549.2807 m/z). Examples of the detection of candicidin in both the standard (**A**) and membrane vesicle samples (**B**) are highlighted. This was performed using both authentic candicidin and antimycin standards and purified MVs from the *S. albus* S4 strains. Examples of the candicidin standard (**C**) and a membrane vesicle sample (**D**) are shown. The values generated for the peak of candicidin (from the extracted ion chromatogram) were plotted below (**E**). No antimycin was detected in any of the MV samples

Candicidin and antimycin can both be identified by their distinct UV–Vis spectra and this methodology has been used previously to verify their presence in biological systems (Seipke et al. 2011). Candicidin (BioAustralis, BIA-C1564) and antimycin (Sigma, A8674) were dissolved in absolute ethanol. The characteristic UV–Vis spectra of both antifungals at 100 µg/mL and 1 mg/mL are shown in (Fig. 4A, B). These spectra highlighted absorption maxima at 339 nm, 358 nm, 378 nm and 400 nm (for candicidin) and 320 nm (for antimycin) when dissolved in ethanol. Vesicles from *S. albus* S4 WT, Δ*antC*, Δ*fscC* and Δ*antC* Δ*fscC* strains were purified using the standard protocol but the MV ‘pellets’ were resuspended directly into butanol instead of HEPES buffer. The MVs were analysed by UV–Vis spectrophotometry and it was clearly shown that vesicles from the WT and Δ*antC* strains gave spectra indicative of candicidin (with no antimycin). Also, vesicles from Δ*fscC* and Δ*antC* Δ*fscC* gave no characteristic spectra of either candicidin or antimycin. These results mirror those obtained previously using the *C. albicans* bioassay (Fig. 3B, C) and show that in *S. albus* S4, candicidin was packaged into MVs whereas antimycin is absent/excluded. Lastly, mass spectrometry was used to confirm the presence of candicidin (but not antimycin) in MVs purified from *S. albus* S4 WT and *ΔantC* strain but not from Δ*fscC* or Δ*antC* Δ*fscC* (Fig. 5, Supplementary Information 8 and Table 1)*.*

**Table 1:** Summary of results from analyses used to detect candicidin and antimycin in *Streptomyces albus* S4 MVs

Strain purified MVs originated from	Is a zone of inhibition produced with *C*. *albicans* in the bioassays?	Is there a UV–Vis spectrum characterstic of candicidin from the MV samples?	Is there a UV–Vis spectrum characterstic of antimycin from the MV samples?	Has candicidin been detected in the MV samples by mass spectromtery?	Has antimycin been detected in the MV samples by mass spectromtery?	Final conclusion
Streptomyces S4 wild type	✓	✓	X	✓	X	Streptomyces S4 WT MVs contain candicidin but not antimycin
Streptomyces S4 ΔantC	✓	✓	X	✓	X	Streptomyces ΔantC MVs contain candicidin but not antimycin
Streptomyces S4 ΔfscC	X	X	X	X	X	Streptomyces ΔfscC MVs contain neither candicidin nor antimycin
Streptomyces S4 ΔantCΔ fscC	X	X	X	X	X	Streptomyces ΔantC ΔfscC MVs contain neither candicidin nor antimycin

The antifungal content of the purified MVs were tested using bioassays with *Candida albicans* (Fig. 3), UV–Vis spectra (Fig. 4) and mass spectrometry (Fig. 5). The results of all these tests are summarised below

## Discussion

To our knowledge, the results of this study provide the first demonstration of the packaging and bioactivity of an antifungal compound (candicidin) in MVs secreted from a natural and non-engineered Gram-positive bacterial strain (*S. albus* S4). Using our understanding of the AmBisome^®^ drug delivery system (Stone et al., 2016) and previous work on *Streptomyces* MVs (Schrempf et al. 2011 and Shrempf and Merling 2015), we had reasoned that *S. albus* S4 bacteria may naturally package antifungal compounds into MVs to facilitate their diffusivity and targeting and to enhance their overall potency against pathogenic fungi such as *C. albicans*. Furthermore, the availability of mutants deficient in one or both of the antifungals (WT, Δ*antC* [candicidin producing], Δ*fscC* [antimycin producing] and Δ*antC* Δ*fscC* [produces neither]) also enabled us to investigate whether the biosynthesis of either candicidin and/or antimycin was specifically linked to MV biogenesis in *S. albus* S4. Our findings show that the biogenesis of MVs in *S. albus* S4 was not reliant upon either candicidin or antimycin synthesis. The results presented in Fig. 1A–H showed that the yield and MV diameter/appearance was unaffected by any mutation (Δ*antC* and/or Δ*fscC),* evidencing that these specific mutations do not impact upon the regulation and biogenesis of MVs.

The use of Wheat Germ Agglutinin (WGA), a lectin from *Triticum vulgaris* (wheat) conjugated to the fluorescent derivative fluorescein isothiocyanate (FITC) allowed us to probe whether the MVs produced by *S. albus* S4 contained peptidoglycan (a major component of the Gram-positive cell membrane). It was clearly demonstrated that peptidoglycan was detected in *S. albus* S4 cells (Fig. 1I–L) but not in the purified MVs (Fig. 1M, N). Although biogenesis of MVs in Gram-positive organisms is still poorly understood, this finding supports the gathering evidence that they are derived from the cytoplasmic membrane and are formed at sites of peptidoglycan weakness or depletion as shown recently in *Staphylococcus aureus* (Wang et al. 2018).

In this study, *S. albus* S4 MVs were found to be enriched with specific proteins when compared to the originator whole cell proteins (Fig. 2A, B). This provides evidence of selective enrichment of protein cargo to the secreted membrane vesicles. Moreover, MVs isolated from the double mutant (Δ*antC* Δ*fscC*) contained a markedly different protein profile when compared with MVs from the WT, Δ*antC* and Δ*fscC* strains. Interestingly, MVs purified from each strain were enriched with a serine protease, which was also absent in the whole cell protein profile (Fig. 2A lanes 4, 6, 8 and 10 compared to 3, 5, 7 and 9). The packaging of a serine endopeptidase within a MV may be beneficial to prevent ‘premature’ hydrolysis of the target substrate(s) by compartmentalisation. Evidence presented in (Fig. 2C, D) provided some support for this as the addition of SDS led to changes in the MV protein profile. In Gram-negative bacteria, SDS is thought to cause disruption of the vesicle membranes (McCaig et al. 2013) and the release of active proteases within, which can cause degradation of other membrane vesicle-associated proteins. This was demonstrated by the disappearance of many (but not all) MV proteins (Fig. 2C lanes 4–6 compared with lane 3).

As outlined previously, we reasoned that the packaging of antifungals may offer some benefit to the producer organism. We were particularly interested to understand whether the disruption of either candicidin or antimycin biosynthesis would also abrogate the yield of MVs as had previously been shown in the study of Hoefler et al. (2017) using *Streptomyces* Sp Mg1 producing the polyene antibiotic linearmycin. The latter was not demonstrated in our studies using *S. albus* S4 (Fig. 1A–H) and further functional studies provided insight into the packaging of antifungals in MVs derived from this strain. A bioassay was developed using authentic candicidin and antimycin and we showed that these ‘unencapsulated’ antifungals were inhibitory to *C. albicans* at concentrations of 10 and 5 µg/mL, respectively (Supplementary Information 5). As expected, zones of inhibition were produced from *S. albus* S4 WT (produces candicidin and antimycin), Δ*antC* (candicidin-producing) and Δ*fscC* (antimycin-producing) strains on a lawn of *C. albicans* with no zone of inhibition from the double mutant (Δ*antC* Δ*fscC*) (Fig. 3A). Next, purified MVs were used in this bioassay and it was clear that candicidin was specifically packaged into the MVs as there were clear zones of inhibition produced from *Streptomyces* WT and Δ*antC* MVs (Fig. 3B). The UV–Vis spectra of the purified MVs (Fig. 4C, D) also confirmed the above findings and also clearly showed the presence of candicidin but not antimycin in these samples. Finally, mass spectrometry data also confirmed the presence of candicidin in MVs from WT and Δ*antC* strains but not in Δ*fscC* or Δ*antC* Δ*fscC* (Fig. 5).

The idea of the selective packaging of candicidin but not antimycin is intriguing and may be informed by an interplay of the following factors. First, there is a difference in the hydrophobicity/lipophilicity of the two compounds, which have very different partition coefficients as judged by LogP (values obtained from PubChem, [National Center for Biotechnology Information 2022a and b]). Antimycin (4.8) is considerably more hydrophobic than candicidin (1.7), possibly aiding the packaging of the latter in the lumen of the MVs as indicated by the retention of activity in the vesicles after washing (Supplementary Information 7). Furthermore, the biosynthesis of the two compounds is distinct, with candicidin akin to linearmycin (Hoefler et al 2017) and amphotericin (formulated in the Ambisome) (Stone et al. 2016), both of which are previously shown to be associated with and/or benefitting from formulation with vesicles either naturally or synthetically. Previous work has also shown that while there is co-ordinate regulation of antimycins and candicidin (McLean et al. 2016), there is no synergy in their activities against *Candida*. It is possible that packaging could be co-ordinately regulated to separately deliver the antifungals as packaged and non-packaged antifungals. Lastly, it is also noted that antimycin is produced during vegetative growth and may be temporally separated from candicidin production which has been shown to be phosphate regulated in *Streptomyces* spp. (Gil and Campelo-Diez 2003). Overall, there are many possible explanations as to why candicidin but not antimycin is packaged into MVs as the two antifungals differ in regulation, synthesis and physicochemical properties. Presently, the exact mechanism of the selection of candicidin (and exclusion of antimycin) into the MVs is unclear and requires further investigation.

In summary, it can be concluded that *S. albus* S4 produces membrane vesicles that are primarily derived from the cytoplasmic membrane, most likely via a process of vesiculation at regions of weakness in the peptidoglycan layer. These MVs appear to be enriched with a range of small (e.g., candicidin) and large (e.g., serine endopeptidase) molecules, while excluding others (e.g., antimycins). Although we still have much to understand about vesiculation in Gram-positive cells, this insight will lead to the increased understanding of biogenesis and targeting in natural systems such as that previously shown in *Streptomyces coelicolor* (Schrempf et al. 2011 and Faddetta et al. 2021), *Streptomyces lividans* (Schrempf and Merling 2015) and *Streptomyces* Sp. Mg1 (Hoefler et al. 2017). Lastly, these findings may also aid the development of cutting-edge pharmaceutical formulations with enhanced uptake of antifungals, such as the synthetic liposomal formulation of Amphotericin B, AmBisome^®^ (Walker et al. 2018).

## Materials and methods

- Microbial cultivation

### Strains

*S. albus* S4 strains (wild type, *ΔantC, ΔfscC* and Δ*antC* Δ*fscC*) were all sourced from Professor Matt Hutchings (John Innes Centre) and Dr Ryan Seipke (University of Leeds). *Candida albicans* SC5314 was sourced from Dr Luisa De Sordi (Assistant Professor at Sorbonne Université).

Media All media were prepared by the addition of components listed to the desired volume of distilled water which were then sterilized by autoclaving. The main media used for bacterial growth were as follows: Lysogeny Broth (LB) 10 g/L Bacto tryptone, 5 g/L Bacto yeast extract, 5 g/L sodium chloride, Tryptone Soy Broth (TSB) 30 g/L TSB pre-made powder, Yeast Extract Malt Extract Medium (YEME) 3 g/L Bacto yeast extract, 3 g/L Malt extract, 5 g/L Bacto peptone, 10 g/L glucose. Agar (Oxoid) was added (20 g/L) to the media before autoclaving for the preparation of agar plates. *S. albus* S4 was cultured on Mannitol Soya (MS) agar plates composed of 20 g/L soya flour (Holland and Barrett), 20 g/L mannitol, 20 g/L agar. The media were autoclaved twice before pouring to ensure there were no spores.

b) MV purification

### Standard protocol for purifying MVs from *S. albus* S4 strains (adapted from Blackburn et al. 2021)

20 μl of spore stock was added to 1 mL sterile ultrapure water (Thermo Scientific™ Barnstead™ Easypure™ II) and spread on to a sterile MS agar plate. The plate was incubated at 30 °C for 5–7 days. Colonies were inoculated into 12.5 mL YEME:TSB media and incubated at 30 °C, 180 RPM for 72 h. 5 mL of each culture was inoculated into 500 mL LB in 2 L baffled flasks then incubated at 30 °C, 180 RPM for 72 h. OD_600_ of each culture was determined using a range of dilutions and confirmed to be approximately 3.0 for each strain. The bacterial culture was pelleted by centrifugation at 5000×*g* for 30 min at 4 °C and the supernatant was removed and filtered through a 0.45 µm polyethersulfone (PES) membrane filter to remove any whole bacterial cells or large bacterial fragments. To ensure that all live bacterial cells had been removed, 500 μL–1 mL of filtered supernatant was spread onto LB agar plates and incubated for 24–48 h at 37 °C to check for growth. MVs were precipitated out of solution by slowly adding 1.5 M ammonium sulphate then incubated overnight at 4 °C with gentle stirring. The MVs were pelleted by centrifugation at 16,000 RPM (25,805 × *g*) for 30 min at 4 °C. The resulting MV pellets were resuspended in 10 mM N-2-hydroxyethylpiperazine-N-ethanesulfonic acid (HEPES)/0.85% NaCl, pH 7.4 then finally filtered again through a 0.45 µm membrane filter.

c) MV characterisation

### Microscopy

#### Transmission electron microscopy protocol to visualise MVs

Membrane vesicles resuspended in HEPES buffer were concentrated for electron microscopy (EM) by centrifugation at 13,200 RPM (14,220×*g*) for 30 min at 4 °C. The MV pellets were resuspended in 10 μL HEPES buffer then added to a formvar/carbon-coated copper EM grid (mesh size 400) and left to settle for 10 min. MVs were then fixed by adding 10 μL of 4% formaldehyde in PBS for 10 min. The grids were subject to 4 × 1 min water washes then negatively stained using 2% uranyl acetate in water. Grids were air dried and loaded on to the Jeol transmission electron microscope (model JEM 1230). Photos taken using Gatan Multiscan/Gatan One View digital cameras and operated at an accelerating voltage of 80 kV.

### Confocal microscopy for *S. albus* S4 cells and MVs

*S. albus* S4 cells were inoculated into 12.5 mL YEME:TSB media and incubated at 30 °C, 180 RPM for 48 h. 1 mL cells were pelleted by centrifugation at 13,200 RPM (14,220×*g*) for 30 min so that both cells and MVs were pelleted together. The supernatant was removed and 1 mL 2% formaldehyde in PBS was used to fix the cells. The cells were pelleted again and 1 mL 100 µg/ml WGA-FITC (Sigma L4895) was mixed with the cells and incubated in the dark for 1 h. 15 μL cells were added onto a 1.5 mm thickness coverslip before being inverted into a drop of ProLong Gold antifade mountant (Life Technologies, P36930) on a glass slide. Slides were incubated at room temperature in the dark overnight to cure. Samples were visualised the next day by confocal microscopy (Zeiss lsm 880 with airscan with associated Zen Black software). A scale bar was added to images using Fiji (Image J). The only modification to the protocol when using *S. albus* S4 MVs was that there was an additional concentration step after the incubation with WGA-FITC for 1 h. In this method, MVs were pelleted by centrifugation at 13,200 RPM (14,220×*g*) for 30 min then resuspended in 15 μL PBS, which was added to the glass slide in the same way as above.

d) Protein manipulation techniques

### Bradford assay

The concentration of protein in cells and MV samples were determined using a Bradford assay. Bradford reagent (Bio-Rad catalog # 5,000,006) was used and the assay was performed as per the manufacturer’s instructions.

### TCA precipitation of MVs

Purified MVs (resuspended in HEPES buffer) were thoroughly mixed with ice-cold 100% trichloroacetic acid (TCA) stock solution (Sigma-Aldrich, catalog #T4885) to make a final concentration of 20% TCA. Samples were incubated on ice for 30 min then centrifuged at 13,200 RPM (14,220×*g*) for 30 min at 4 °C. The supernatant was removed and the pellet was resuspended in 0.5 mL ice-cold acetone. The samples were centrifuged at 13,200 RPM (14,220×*g*) for 15 min at 4 °C. The supernatant was removed and each pellet was resuspended in HEPES buffer.

### SDS-PAGE

Samples were standardised to the same protein concentration then subject to TCA precipitation to concentrate. Each sample was then mixed with the appropriate volume of 4 × RSB (Reducing Sample Buffer, Invitrogen catalog #NP0008) and heated to 95 °C for 5 min. SDS-PAGE (sodium dodecyl sulphate–polyacrylamide gel electrophoresis) gels were run using the Invitrogen Novex Xcell II Mini-Cell system for Electrophoresis with NuPAGE pre-cast 10 well 4–12% Bis–Tris gels. 20 μL of each sample was loaded into each well. 0.2 μL markers (Bio-Rad, catalog #1,610,374) were used each time to estimate protein size. Gels were run at 165 V for 48 min in MES [2-(N-morpholino) ethanesulfonic acid] running buffer or 55 min in MOPS [3-(N-morpholino) propanesulfonic acid] running buffer. SDS-PAGE gels were developed using the Pierce Silver Stain kit (Thermo-Fisher catalog #24,612) as described in the manufacturer’s protocol. Densitometry analyses were performed using Fiji (Image J).

### *S. albus* S4 detergent studies

Various concentrations of SDS (0.1, 0.5 and 1% diluted in sterile ultrapure water) were added to *S. albus* S4 MVs then incubated for 60 min at 37 °C, shaking at 180 RPM. Samples were TCA precipitated and finally resuspended in 30 μL HEPES buffer and 10 μl 4 × RSB prior to loading on to an SDS-PAGE gel.

- e) Detection of antifungal compounds in *S. albus* S4 MVs

### Preparation of *S. albus* S4 cells for *C. albicans* bioassay

*S. albus* S4 colonies (from MS plates) were inoculated into TSB:YEME (1:1) and incubated at 30 °C, 180 RPM for 72 h.  μL of this culture was spotted at the centre of an LB agar plate and left to soak/dry for 2 h. Plates were incubated at 30 °C for 72 h before *C. albicans* addition.

### Preparation of candicidin and antimycin for *C. albicans* bioassay

Candicidin (BioAustralis, BIA-C1564) and antimycin (Sigma, A8674) were resuspended in ethanol and diluted to give various concentrations ranging from 1 mg/mL to 1 µg/mL. 10 μL candicidin/antimycin at each concentration were added to LB plates and left to soak/dry for a minimum of 2 h at room temperature and 10 μL ethanol only was used as a negative control.

### Preparation of *S. albus* S4 MVs for *C. albicans* bioassay

5–10 μL purified MVs were added to LB plates and left to soak/dry for a minimum of 2 h at room temperature.

### *C. albicans* addition to plate

*C. albicans* colonies were inoculated into YPD media and incubated at 37 °C for 18–24 h then diluted to an OD_600_ of 1.0. Cells were centrifuged at 5000 RPM (2800×*g*) for 5 min and the supernatant was discarded. *C. albicans* cell pellets were resuspended into 50 mL ‘hand hot’ LB agar (0.5% w/v) then 10 mL was slowly added to each plate. Plates were incubated at 37 °C for 18 h and the diameter of the zone of inhibition was measured. All plates were prepared in triplicate so that the average zone of inhibition and standard deviation was calculated for MVs from each strain.

### UV–Vis spectra of *Streptomyces* MVs

The spectrophotometer Agilent Technologies Cary 60 UV–Vis was used to detect the UV–Vis spectrum of candicidin and antimycin. 500 μL samples were loaded on to the spectrophotometer in UV quartz cuvettes.

### Preparation of MVs for mass spectrometry

*S. albus* S4 MVs were purified from all four strains using the standard protocol and the final MV pellet was resuspended in 2 mL HEPES buffer and filter sterilised (0.45 µm pore size). The MVs were washed 3 × using 2 mL HEPES buffer and centrifugations at 13,200 RPM (14,220×*g*) for 30 min at 4 °C. The final MV pellet was resuspended in 2 mL HEPES buffer then mixed with 2 mL butanol and left for 15 min to settle. The top layer was extracted and the butanol was evaporated using a vacuum centrifuge. The final pellet was resuspended in 20 µL 50% (v/v) methanol in ultrapure water and a 4 µL injection volume was used for each sample run. The candicidin and antimycin standards were used at 100 µg/mL (w/v) concentration in 50% (v/v) methanol and a 0.5 µL injection volume was used for each sample run.

### Mass spectrometry

Purified vesicles were analysed by ultra-performance nanoLC (ACQUITY M Class, Waters) coupled to an IMS mass spectrometer (SYNAPT G2-Si, Waters) fitted with a NanoLockSpray source (Waters). Samples were loaded via a Symmetry C18 5 µm, 180 µm × 20 mm trap column (Waters) and separated through a HSS T3 C18 1.8 µm, 75 µm × 150 mm analytical column (Waters). Samples were separated using a 5 to 95% acetonitrile 0.1% formic acid gradient over 35 min at a flow rate of 300 nL/min. The mass spectrometer was operated in positive ion mode with a capillary voltage of 3.0 kV, cone voltage of 40 V and a source offset of 80 V. Before analysis the instrument was calibrated with NaI and during analysis a LockMass reference, Glu-1-fibrinopeptide B (Waters), was delivered to the NanoLockSpray source. Mass spectra were collected, over 50–2000 m/z, alternating between low (4 eV) and elevated (15–45 eV) collision energies at a scan speed of 1.0 s. Data were processed using the UNIFI software package from Waters (Wilmslow, U.K.).

## Supplementary Information

Below is the link to the electronic supplementary material.

Supplementary file1 (DOCX 8158 KB)
