# Supplementary material for: The polyene antifungal candicidin is selectively packaged into membrane vesicles in Streptomyces S4
Source: Arch Microbiol. 2022 Apr 30;204(5):289. doi: 10.1007/s00203-022-02906-w (PMC9054904; doi:10.1007/s00203-022-02906-w)
Supplement: Supplementary file 1 — Supplementary file1 (DOCX 8158 KB) [file 203_2022_2906_MOESM1_ESM.docx]

# SUPPLEMENTARY DATA for:

# The Polyene Antifungal Candicidin is Selectively Packaged into Membrane Vesicles in *Streptomyces* S4.

*Submitted to Archives of Microbiology*

## Sarah A. Blackburn^1^, Mark Shepherd^2^ and Gary K. Robinson^3^

^1,2,3^ – School of Biosciences, Division of Natural Sciences, University of Kent, Canterbury, CT2 7NJ.

^3^ – Corresponding author email address – [G.K.Robinson@kent.ac.uk](mailto:G.K.Robinson@kent.ac.uk)

Orcid numbers

^1^- 0000-0002-7504-103X

^2^ - **0000-0002-7472-2300**

**^3^-** 0000-0003-2660-7778

**Supplementary Information 1**

Professor Hutchings (UEA) kindly donated the following strains for study into MV production in *Streptomyces albus* S4.

**A brief introduction and summary of the *Streptomyces albus* S4 strains used in this study**

**Supplementary Information 2**

**Comparison of *Streptomyces* *albus* S4 MVs to those isolated from *S. lividans*, *E. coli* and *P. aeruginosa***
TEM images of purified MVs from the following *Streptomyces* *albus* S4 strains: *Streptomyces* S4 WT **(A),** *Streptomyces ΔantC* **(B)***, Streptomyces ΔfscC* **(C)***,* *Streptomyces ΔantC ΔfscC* **(D)***.* Images **E-F** were sourced and cropped from Figure 1, Schrempf *et al.* 2015. MVs were obtained after culturing *S. lividans* on an agar-containing medium for 7 days**.** Own TEM images of purified OMVs from *Pseudomonas aeruginosa PA01* **(G)**, *PA14* **(H)** and *E. coli* BL21 (DE3) **(I)**.

**
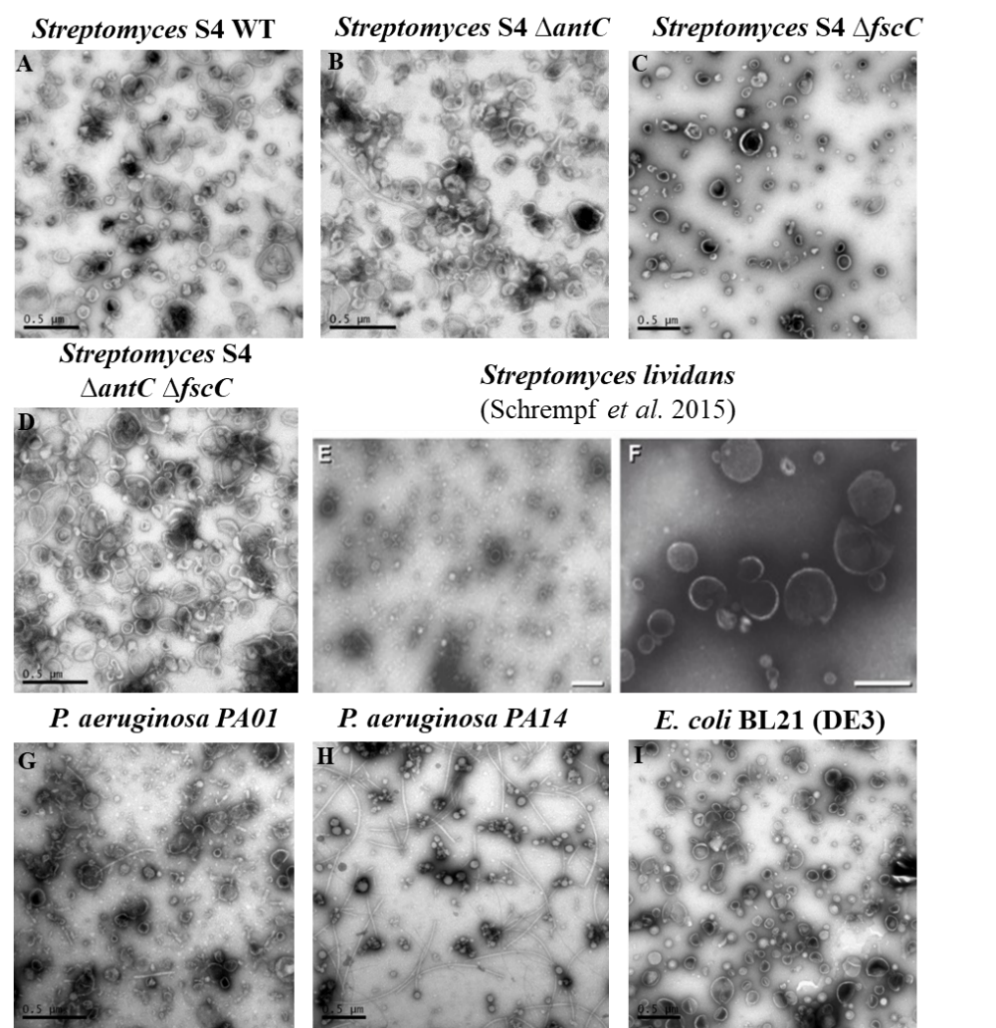
**

**Supplementary Information 3**

**Visualisation of *Streptomyces albus* S4 MVs using WGA-FITC.**

MVs were purified from the following strains: *Streptomyces albus* S4 WT **(A-B)**, *ΔantC* **(C)**, *ΔfscC* **(D)** and *ΔantC ΔfscC* **(E)**. MVs were concentrated by centrifugation at 13,200 RPM (14,220 × *g*) for 30 minutes. MVs were fixed in 2% (w/v) formaldehyde then incubated in 100 µg/mL WGA-FITC (Sigma L4895) in the dark for 1 hour. MVs were pelleted by centrifugation at 13,200 RPM (14,220 × *g*) for 30 minutes then resuspended in 15 μL PBS. This was added onto a coverslip before being inverted into a drop of ProLong Gold antifade mountant on a glass slide. Samples were visualised the next day by confocal microscopy (Zeiss lsm 880 with airscan) and scale bars were added to images using Fiji (Image J).


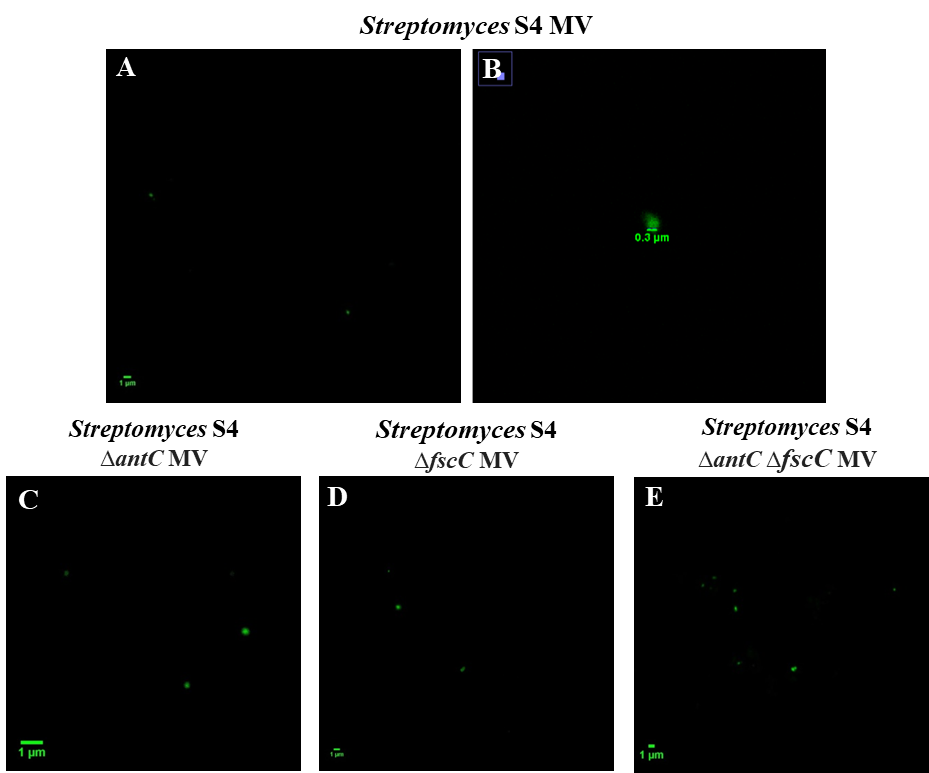


**Supplementary Information 4**

**Comparison of MV protein profiles isolated from *Streptomyces albus* S4 and the 3 mutant strains (∆*antC,* ∆*fscC* and ∆*antC* ∆*fscC*)*.***

MVs were purified from four different *Streptomyces* *albus* S4 strains: WT, ∆*antC,* ∆*fscC* and ∆*antC* ∆*fscC.* A Bradford assay was performed and all samples were standardised to be the same protein concentration. TCA precipitation was used to concentrate samples prior to loading on an SDS-PAGE gel. The SDS-PAGE gel was run then silver stained to visualise the MV protein profile **(A)**. The highlighted band was extracted and identified by mass spectrometry **(B).**

**
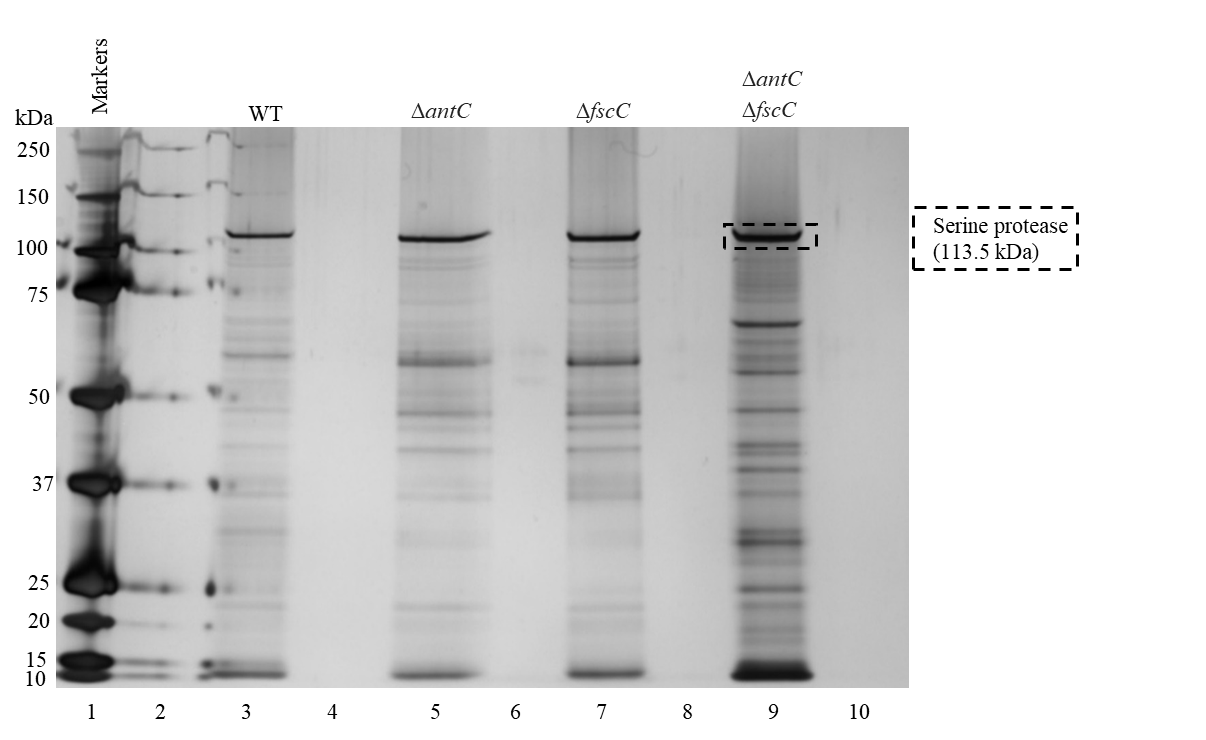
**

**A**

**B**

**Supplementary Information 5**

**The effect of varying concentrations of candicidin and antimycin on *Candida albicans* growth**

Candicidin and antimycin were resuspended in ethanol then diluted to give various concentrations ranging from 200 µg/mL-1 µg/mL. 10 μL candicidin/antimycin was added to LB plates and left to soak/dry for a minimum of 2 hours at room temperature. 10 μL ethanol only was used as a negative control. This was then overlayed with *C. albicans* mixed with LB 0.5% (w/v) agar and left to set. Plates were incubated at 37°C for 18 hours and the diameters of the zone of inhibitions were measured **(A-B)**. All plates were prepared in triplicate and the average zone of inhibition was calculated and presented as a graph **(C-D)**.


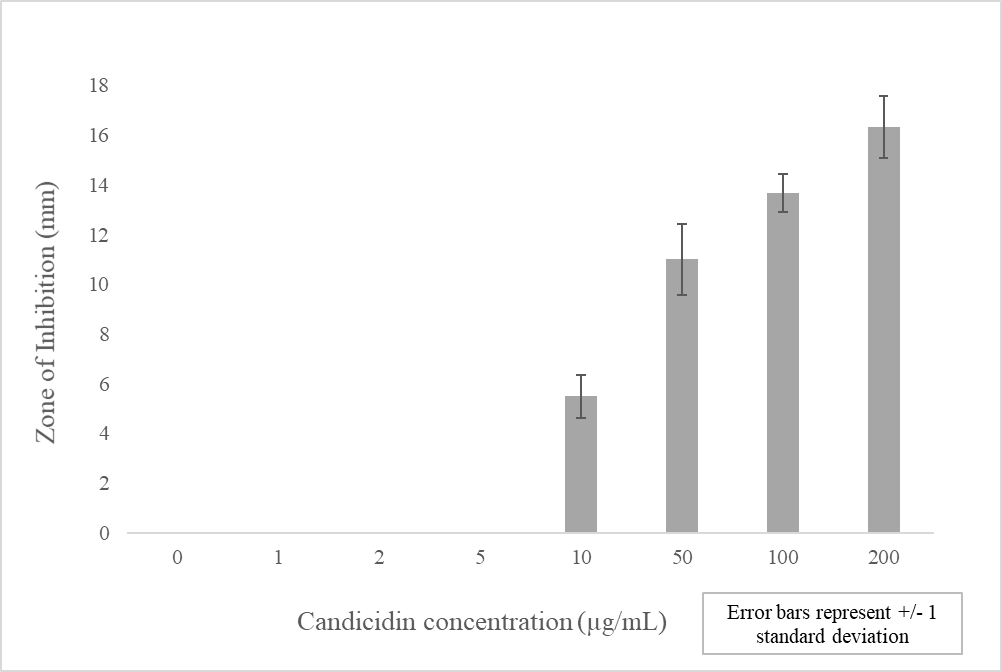

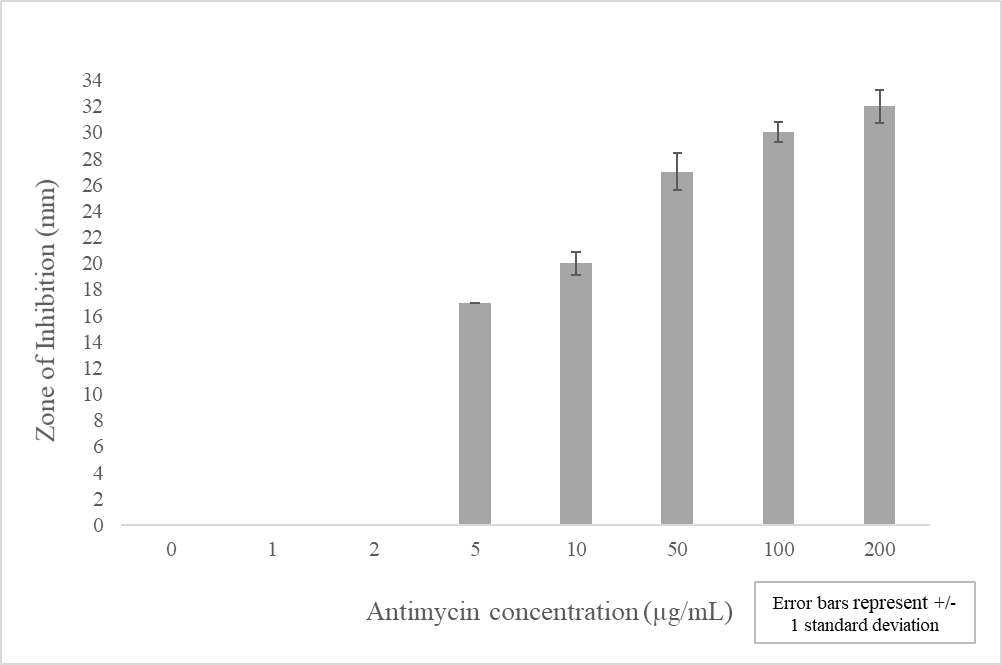

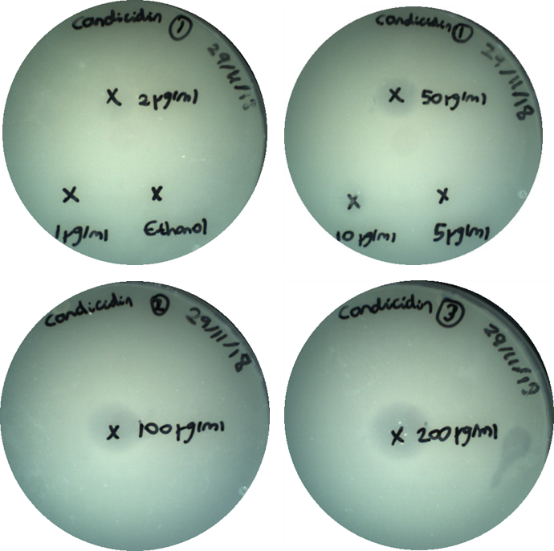

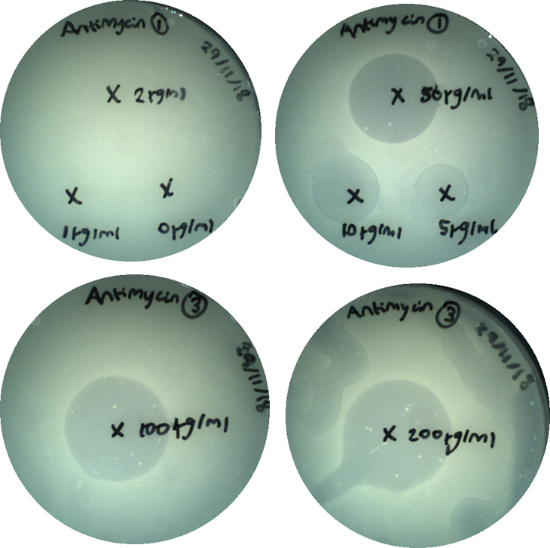


**A**

**B**

**C**

**D**

**Supplementary Information 6**

**The effect of wash steps and MV concentration on the zone of inhibition produced with *Candida albicans***

*Streptomyces albus* S4 MVs were purified and treated in 3 different ways to observe the effect on the zones of inhibition on a lawn of *C. albicans*. *Streptomyces* MV pellets were resuspended in 5 mL 10 mM HEPES buffer and filter sterilised (0.45 µm pore size) as part of the MV purification procedure. 5 μL of this sample was added to LB agar plates and were labelled as the ‘MVs dilute’ sample. The MVs were then concentrated 25x by pelleting 1 mL MVs then resuspending in 40 µL 10 mM HEPES buffer to give a ‘MV 25x concentrated’ sample. Lastly, the MVs were pelleted and washed 3x in 10 mM HEPES buffer to remove anything that was not MV-associated. All centrifugation steps were performed at 13,200 RPM (14,220 x *g*) for 30 minutes at 4°C **(A).** All plates were prepared in triplicate **(B)** and the average zone of inhibition was calculated **(C)**.

**A**

**
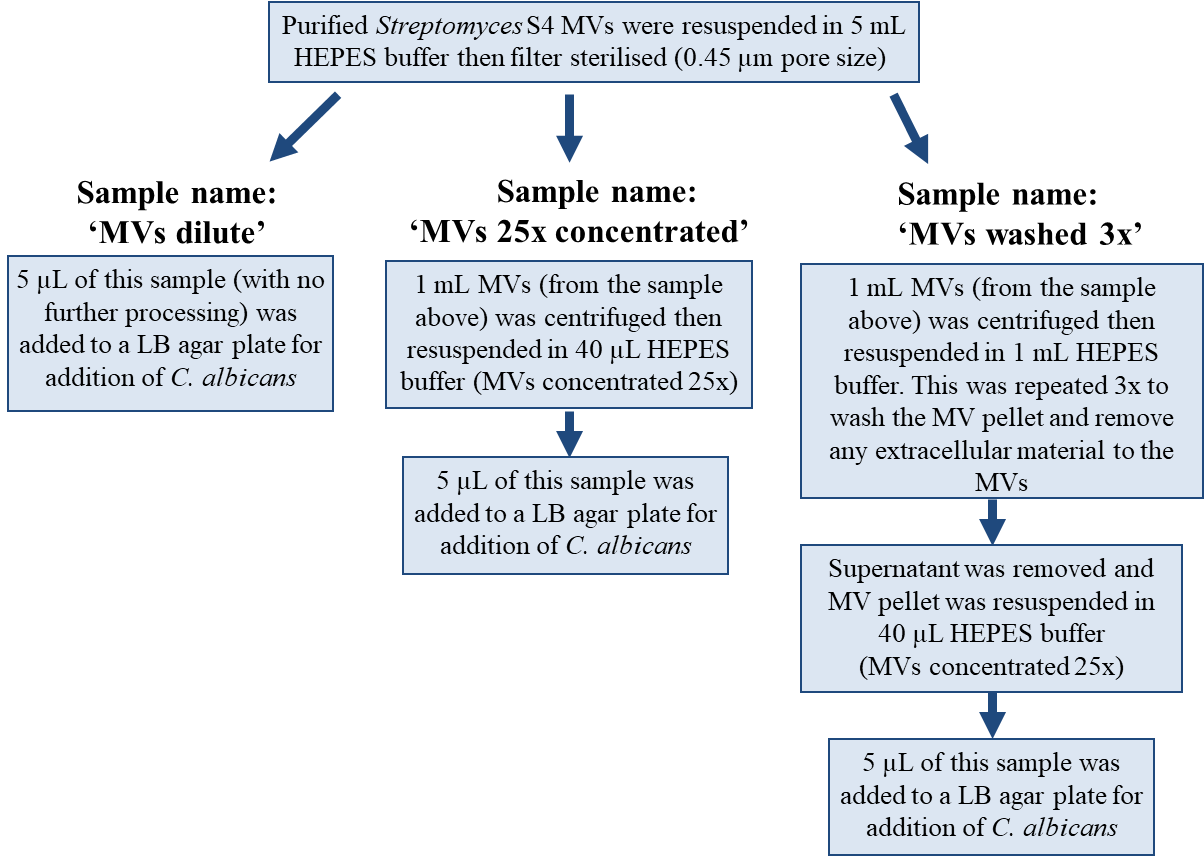
**

**C**


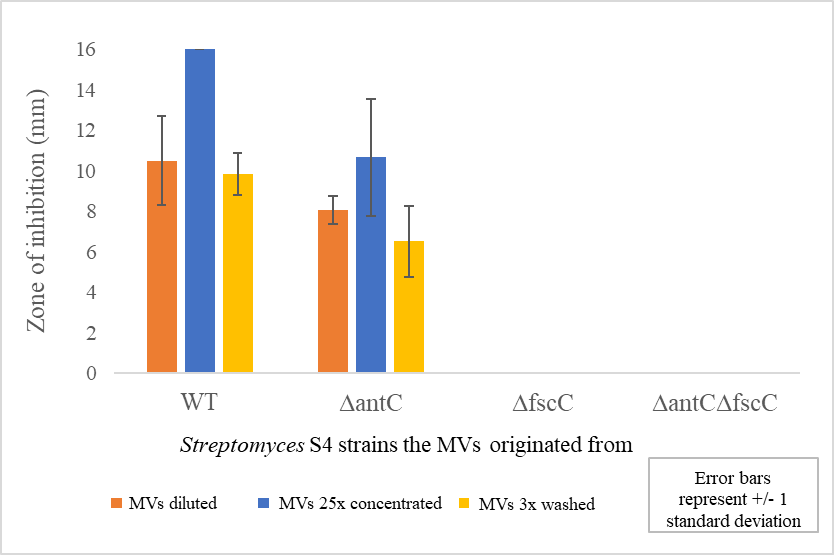


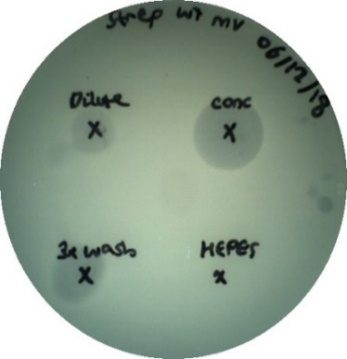


**B**

**Supplementary Information 7**

**Preparation of MVs with various numbers of wash steps to compare the zones of inhibition produced on a lawn of *Candida albicans***

*Streptomyces albus* S4 MVs were washed up to 3x in 10 mM HEPES buffer to observe the effect on the zones of inhibition produced with *C. albicans*. 5 μL of the following MV samples were added to the LB plates and were named ‘MV Dilute’, ‘MV Concentrated’, ‘MV washed 1x’, ‘MV washed 2x’ and ‘MV washed 3x’ as well as 5 μL of their corresponding supernatants (protocols summarised in **A**). All centrifugation steps were performed at 13,200 RPM (14,220 x *g*) for 30 minutes at 4°C. *C. albicans* was mixed with LB 0.5% (w/v) agar then was overlayed on to the plates and left to set. Plates were incubated at 37°C for 18 hours and the diameter of the zone of inhibition was measured **(B)**. All plates were prepared in triplicate and the average zone of inhibition was calculated **(C).**

**
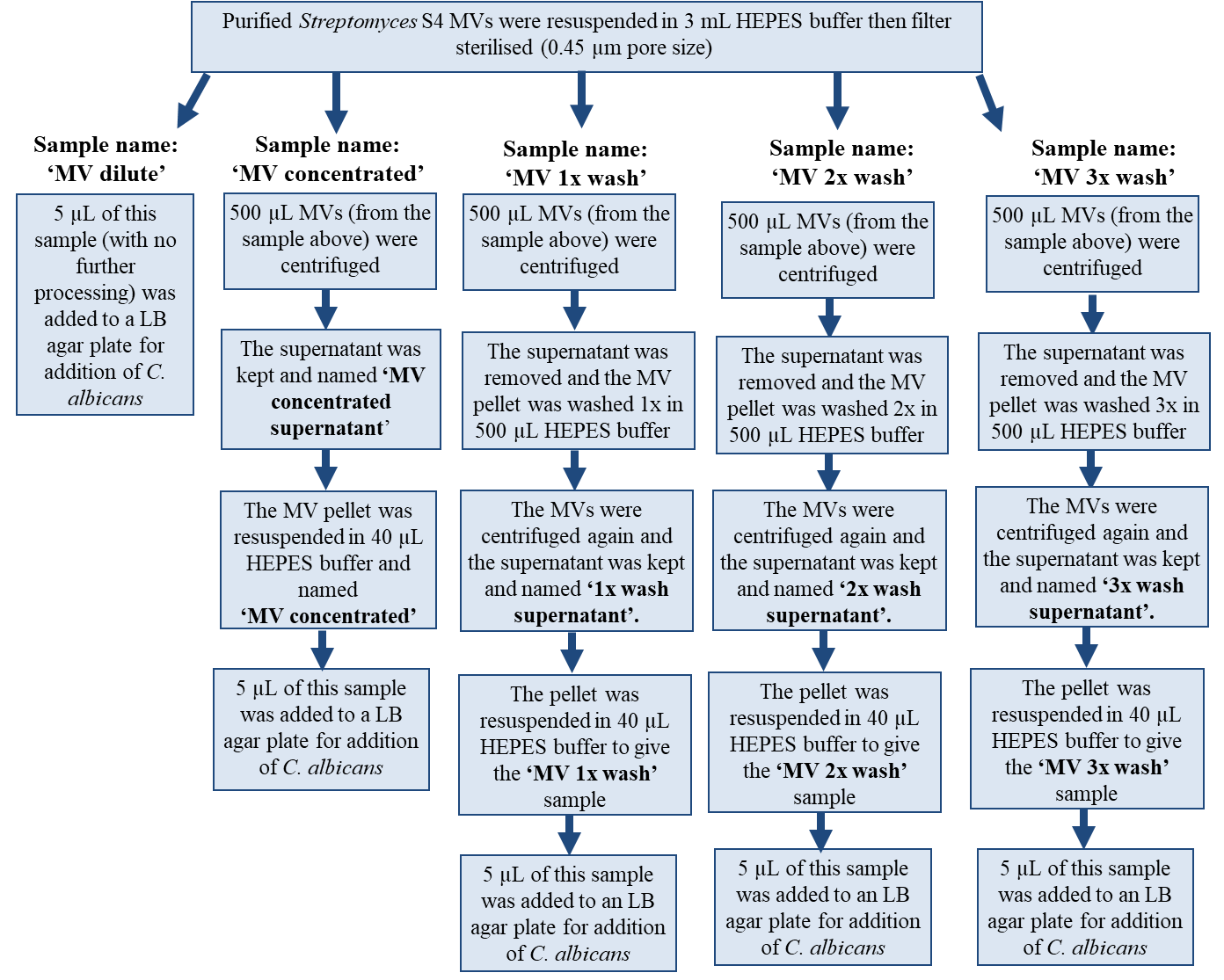
**

**A**

**B**

***Streptomyces* S4 MVs**

***Streptomyces* S4 MV supernatants**


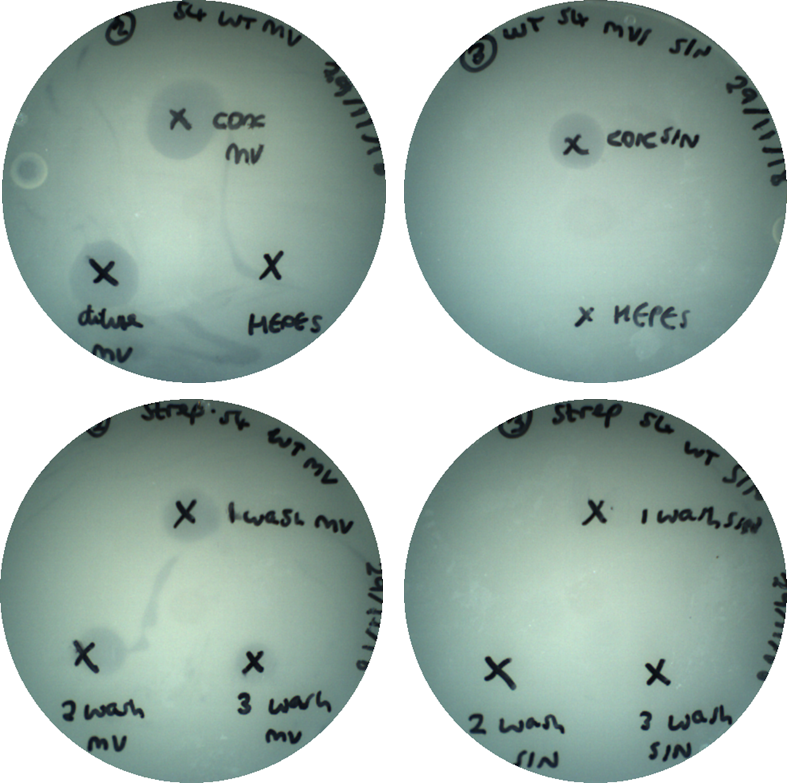

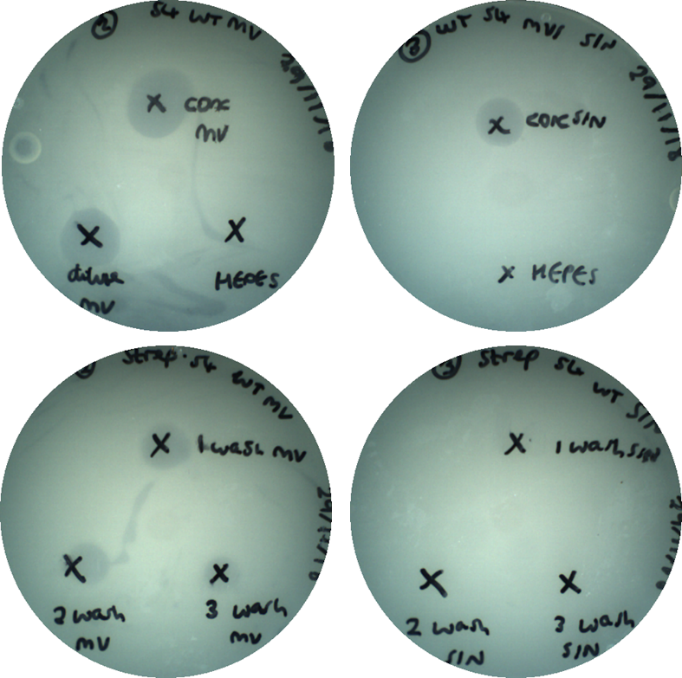

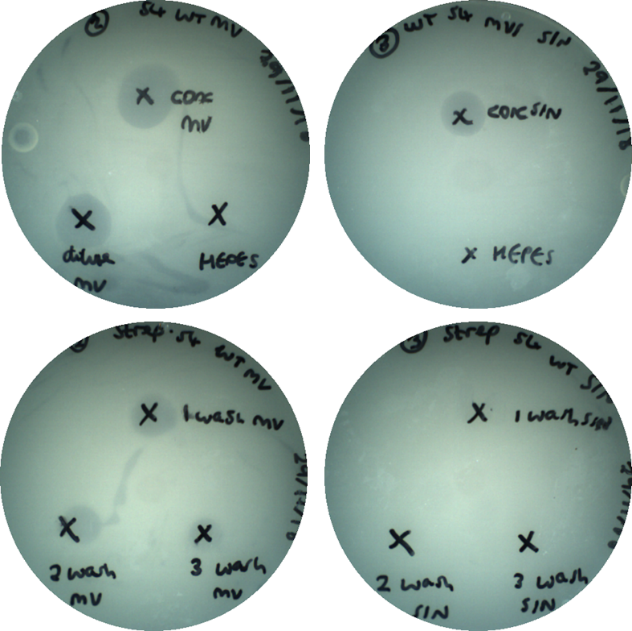

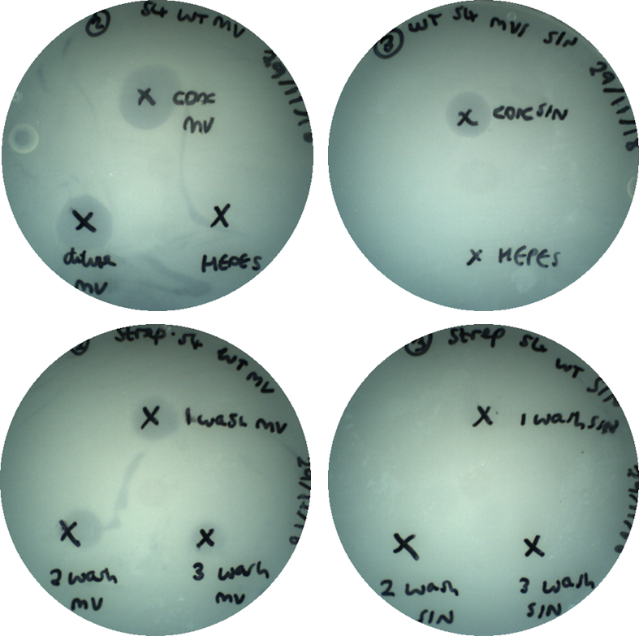


**C**

**
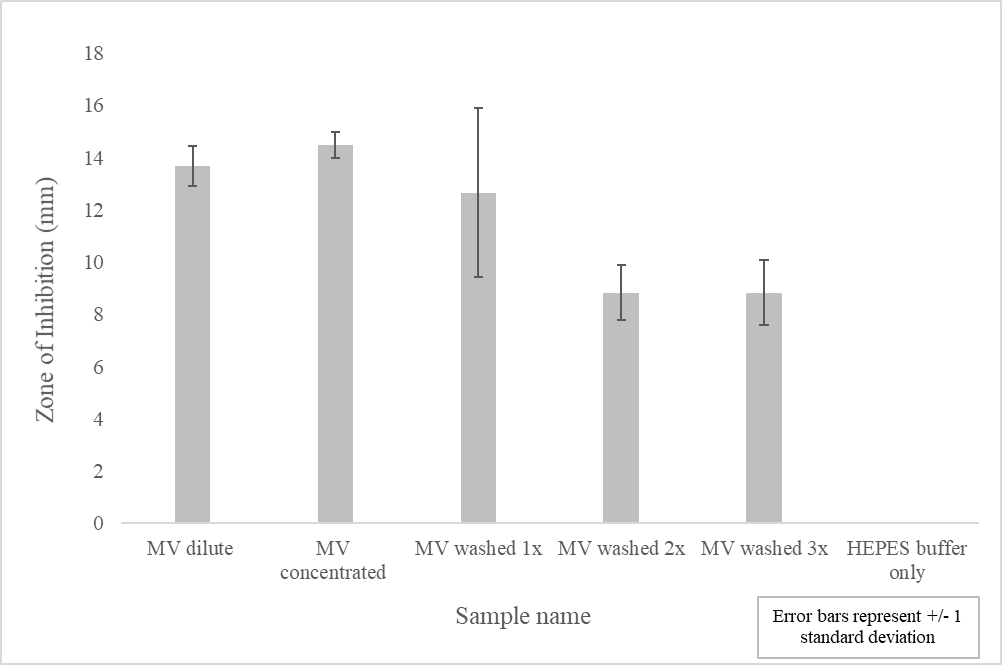
**


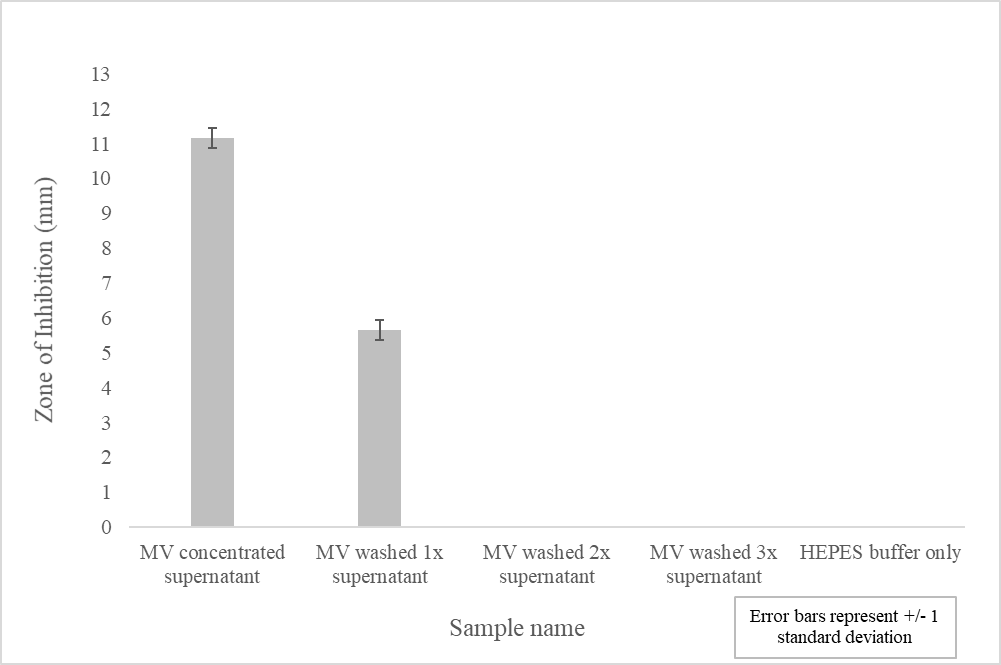


**Supplementary Information 8**

**Confirmation of candicidin present in *Streptomyces* *albus* S4 membrane vesicles using mass spectrometry**

Purified vesicles were analysed by ultra-performance nanoLC (ACQUITY M Class) coupled to an IMS mass spectrometer (SYNAPT G2-Si, Waters) fitted with a NanoLockSpray source. Data was processed using the UNIFI software. Examples of the detection of candicidin in both the standard (**A**) and membrane vesicle samples (**B**) are highlighted. Extracted ion chromatograms were produced to select for the detection of candicidin (EIC [M+H]^+^, 1109.5720 m/z).

**A**

Candicidin standard (BioAustralis)

**
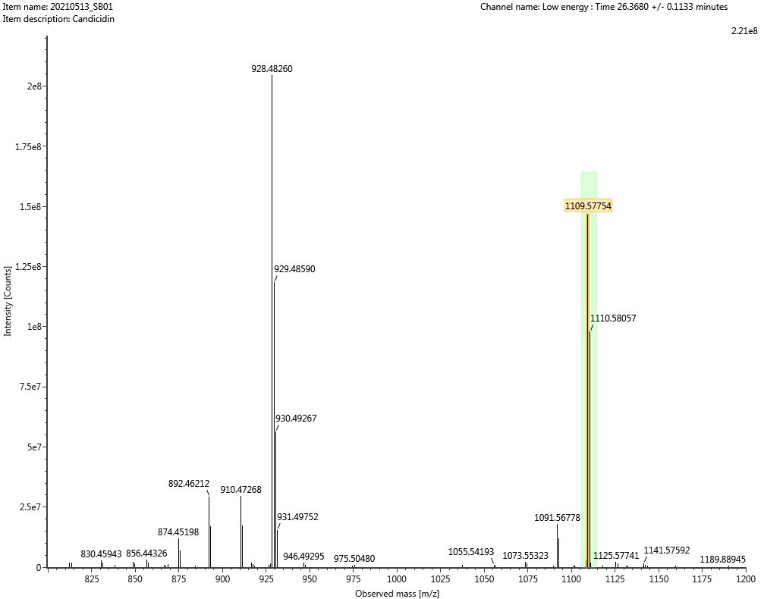

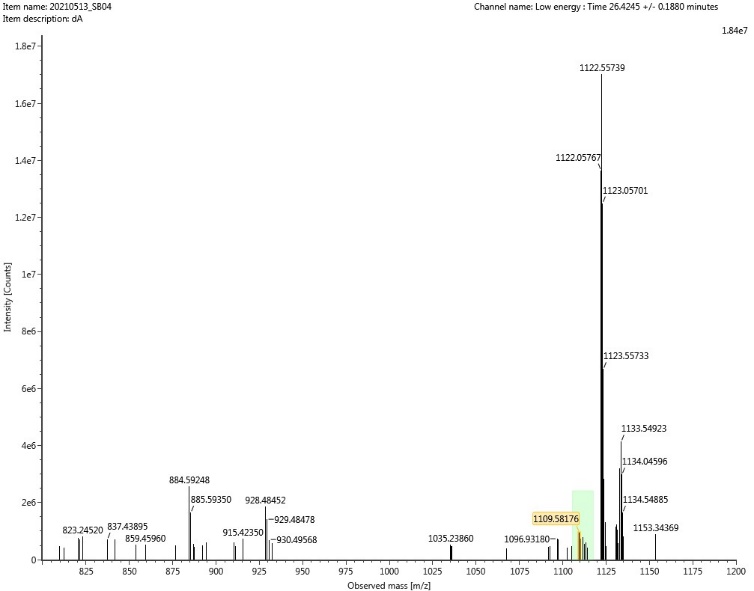
**

**B**

*Streptomyces ∆antC* membrane vesicles
